# A Custom-Made Semiautomatic Analysis of Retinal Nonperfusion Areas After Dexamethasone for Diabetic Macular Edema

**DOI:** 10.1167/tvst.9.7.13

**Published:** 2020-06-11

**Authors:** Lisa Toto, Rossella D'Aloisio, Antonio Maria Chiarelli, Luca Di Antonio, Federica Evangelista, Giada D'Onofrio, Arcangelo Merla, Mariacristina Parravano, Guido Di Marzio, Rodolfo Mastropasqua

**Affiliations:** 1 Ophthalmology Clinic, Department of Medicine and Science of Ageing, University G. D'Annunzio Chieti-Pescara, Chieti, via dei Vestini 31, 66100, Italy; 2 Department of Neuroscience, Imaging, and Clinical Sciences, University G. D'Annunzio Chieti-Pescara, Chieti, via dei Vestini 31, 66100, Italy; 3 IRCCS – Fondazione Bietti, Rome, via Livenza 3, 00198, Italy; 4 Institute of Ophthalmology, University of Modena and Reggio Emilia, Modena, Via del Pozzo 71, 41125, Italy

**Keywords:** retinal nonperfusion areas, intravitreal dexamethasone implant, diabetic macular edema, widefield OCT angiography

## Abstract

**Purpose:**

To evaluate the changes of retinal capillary nonperfusion areas and retinal capillary vessel density of the superficial capillary plexus (SCP) and deep capillary plexus in patients with diabetes with diabetic macular edema treated with an intravitreal dexamethasone implant (IDI).

**Methods:**

We enrolled 28 patients with diabetic retinopathy and diabetic macular edema candidates to IDI. All patients underwent widefield optical coherence tomography angiography with PLEX Elite 9000 device with 15 × 9 mm scans centered on the foveal center at baseline, 1 month, 2 months, and 4 months after IDI. In all the patients, the variation of the retinal capillary nonperfusion areas and of the retinal vessel density of the SCP and deep capillary plexus were calculated using an automatic software written in Matlab (MathWorks, Natick, MA).

**Results:**

During follow-up, SCP showed a statistically significant reduction of ischemic areas at 1 month after IDI (*P* = 0.04) and slightly increased not significantly thereafter (*P* = 0.15). The percentage of nonperfusion areas changed from 11.4% at baseline, to 6.3% at 1 month, 8.1%, at 2 months, and 10.2% at 4 months. The whole vessel density of SCP slightly increased (not significantly) from 35.30% at baseline to 38.00% at 1 month, and then decreased to 37.85% at 2 months and 36.04% at 4 months (*P* = 0.29). Retinal capillary nonperfusion areas and retinal vessel density at the deep capillary plexus did not change significantly (*P* = 0.31 and *P* = 0.73, respectively).

**Conclusions:**

Widefield optical coherence tomography angiography showed a decrease in retinal capillary nonperfusion areas after dexamethasone implant suggesting a possible drug-related reperfusion of retinal capillaries particularly evident in the early period.

**Translational Relevance:**

A custom-made automatic analysis of retinal nonperfusion areas may allow a better and precise evaluation of ischemic changes after intravitreal therapy.

## Introduction

Diabetic retinopathy (DR) is a leading cause of visual loss in working-age populations with an estimated prevalence of any form of DR of 34.6% and proliferative DR (PDR) of 6.96% in the diabetic population. Diabetic macular edema (DME) affects central vision at any stage of DR occurring in 6.81% of patients with diabetes.^1^

Several studies demonstrated the importance of retinal ischemia detection in DR as a predictor of progression of the disease. Increased risk of DR progression was associated with greater involvement of the peripheral retina in terms of retinal ischemia and neovascularization.^2^^,^^3^

Fluorescein angiography (FA) is an essential diagnostic tool in DR staging and can identify both primary vascular lesions and particularly retinal nonperfusion related to neovascularization.^4^ Recently, the introduction of ultra-widefield FA allowed to better investigate peripheral areas of nonperfusion and neovascularization that were missed with previous standard field imaging techniques and to establish correlation between peripheral and central ischemia and between retinal ischemia and DME.^5^^,^^6^

In addition, changes of retinal ischemia after intravitreal treatment either were assessed using anti-vascular endothelial factor (VEGF) or steroids was assessed.^7^^–^^11^

Optical coherence tomography angiography (OCTA) offers major advantages compared with FA, including faster acquisition times, depth-resolved retinal layer information, and the lack of invasive dye administration.^12^

The first studies using conventional OCTA have focused their attention on retinal perfusion parameters of the macular area. They reported a decrease in vascular density of the superficial and the deep capillary vascular network in patients with diabetes with or without DR compared with healthy patients with decreasing values at increasing DR severity.^13^^–^^17^

However, a significant limitation of OCTA has always been a small field of view not allowing exploration of retinal periphery.

For this reason, the introduction of widefield OCTA (WFOCTA) is an important step forward especially for the study of retinal vascular disease.

Some studies have investigated peripheral retina in diabetic eyes to asses retinal capillary nonperfusion and perfusion density using WFOCTA.^17^^–^^20^ In addition, reversal of peripheral ischemia after intravitreal anti-VEGF treatment was investigated in DR using WFOCTA.^21^

The aim of this study is to investigate the changes of retinal capillary nonperfusion areas and whole retinal vessel density of superficial capillary plexus (SCP) and deep capillary plexus (DCP) in patients with DR complicated by DME after intravitreal dexamethasone implant (IDI) using WFOCTA.

## Methods

### Study Participants

In this prospective observational study, 28 eyes of 28 patients with diabetes suffering from DR, classified according to the simplified version of the Early Treatment Diabetic Retinopathy Study classification of the American Academy of Ophthalmology Guidelines Committee, complicated by center-involved DME and candidates to IDI were recruited in the period between December 2018 and May 2019 at our retina center of the Ophthalmology Clinic of University G. D'Annunzio, Chieti-Pescara, Italy. The diagnosis of DR was established by means of retinal fundus examination, FA, and spectral domain OCT.^22^

The study adhered to the tenets of the Declaration of Helsinki and was approved by our Institutional Review Board. Written informed consent was obtained from all participants of the study.

Criteria for inclusion were (1) age greater than 18 years old, (2) diagnosis of diabetes mellitus with DR complicated with DME, and (3) a central macular thickness of greater than 300 micron.

The exclusion criteria were (1) previous intravitreal injections of anti-VEGF or dexamethasone implant in the study eye, (2) diagnosis of glaucoma or ocular hypertension (intraocular pressure of ≥21 mm Hg) at baseline (T0) evaluation, (4) significant media opacities (cataract, vitreous hemorrhage), and (5) inflammatory diseases such as uveitis or retinal vascular occlusion, which may cause macular edema.

### Study Protocol

All patients underwent a complete ophthalmologic examination, including best-corrected visual acuity, intraocular pressure reading, slit-lamp biomicroscopic evaluation, and dilated fundoscopic examination.

All examinations were performed at T0 and at 1 month (T1), 2 months (T2), and 4 months (T3) after the dexamethasone implantation.

Moreover, in all enrolled patients OCTA was performed using widefield PLEX Elite 9000 device (Carl Zeiss Meditec Inc., Dublin, CA) at all time points.

### Workflow Protocol

#### Imaging Protocol: Graders Review Quality, Segmentation, and Artifacts Correction

All eyes were scanned with the PLEX Elite 9000 device (Carl Zeiss Meditec Inc.) that is able to acquire 100,000 A-scans per second with an axial resolution of about 5 µm in tissue, and with a lateral resolution at the retinal surface at about 14 µm.

This device uses a swept laser source which as a central wavelength of 1050 nm (1000–1100 nm full bandwidth).^23^

For each eye, WFOCTA volumes covering a 15x9 mm retinal area and centered at the fovea were acquired at each time point.

During image acquisition, a FastTrack motion correction software was used.

Poor quality images showing a signal strength index lower than 8 and with relevant motion or tilt artifacts were excluded from the analysis and repeated.

All participants’ eyes were imaged three times each, and the best quality image was chosen to be investigated in the study.

Severe tilt artifacts were excluded directly from the study by the two retinal specialists (LT and RDA) adjusting the system's working distance until good signal strength and good alignment between the beam pivot and pupil plane were observed throughout the B-scan.

All selected images were carefully analyzed by the two retinal specialist independently to verify the correctness of segmentation as previously described.^18^

If segmentation errors were detected, the two trained observers performed a manual correction using segmentation and propagation editing tools of the device.

To identify and quantify in detail the main outcome measures all WFOCTA images (field of view of 9 mm × 15 mm, pixel resolution of 0.015 mm) were segmented at the SCP and DCP levels using automatic segmentation by PLEX Elite 9000 device to define the two capillary plexuses.

#### Semiautomated Nonperfusion Analysis

**Figure 1.**
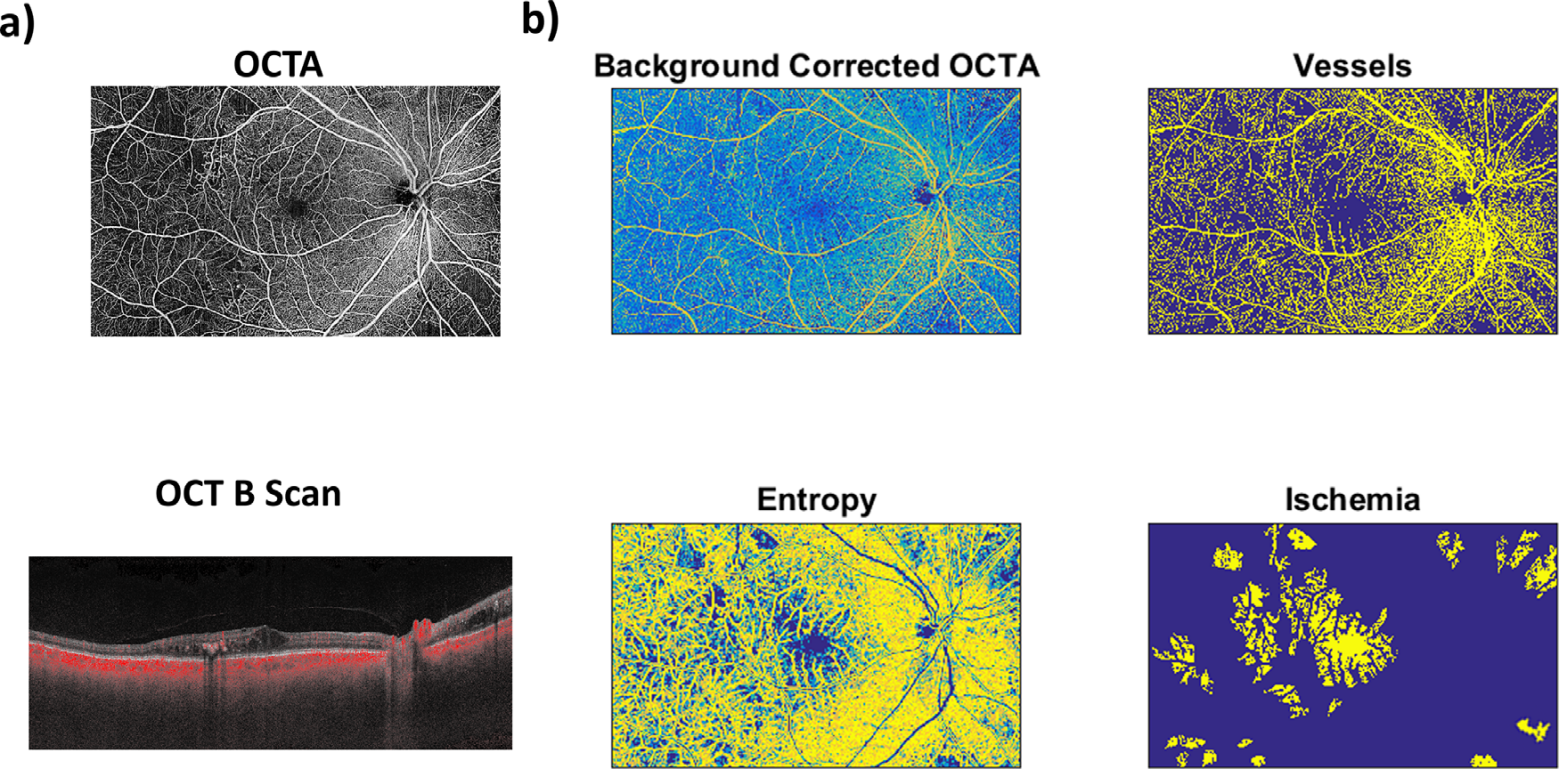
(a) Raw OCTA image and the corresponded OCT B-scan. (b) Processed OCTA images.

A custom-made semiautomatic software, written in Matlab (MathWorks, Natick, MA), was used to identify vessels within the WFOCTA images and to infer regions of ischemia. The algorithm proceeded in five *steps* (Fig. 1).

In the first step, each raw WFOCTA image was low-pass filtered based on a two-dimensional gaussian smoothing kernel with standard deviation of 5 mm (350 pixels).^24^

In the second step, the smoothed image was subtracted from the raw image to obtain a high-pass filtered WFOCTA where slow changing intensity, supposedly related to instrumental and image reconstruction noise, was automatically corrected.^25^

Notice that this preprocessing step assumes a single region of ischemia to be of an area below 350 pixel × 350 pixel, or 5 mm^2^, and it was necessary due to inherent differences in the WFOCTA image contrast as a function of location in space that required algorithmic correction.

In the third step, the processed image was normalized (between 0 and 1, considering its minimum and its 95th percentile value) and thresholded (above 0.5) to create a binary image that highlighted vessels (vessel image). To identify regions of ischemia, the variability in the contrast of the image where vessels are present was exploited.^26^

In the fourth step, a texture-based approach was used by computing the metric of entropy within small regions of 5 pixel × 5 pixel, that is, 0.375 mm^2^. The metric of entropy tends to be high in regions where the thresholded image has both high pixel values (i.e., vessels) and low pixel values (i.e., tissue) and thus it is suited to separate perfused from unperfused regions.

In the fifth and last step, the regions of ischemia where identified using the entropy image, after being masked based on the vessel image, through a thresholding approach where pixels with entropy below 0.3 were deemed as possibly ischemic. Clusters of pixels with low entropy (<0.3) were finally identified as ischemic regions only if they covered an area above 20 pixel × 20 pixel, that is, 0.09 mm^2^.^20^

#### Graders Review Analysis and Edit as Needed

The final outcome of the algorithm was carefully checked and corrected (if needed) by the two independent and highly trained ophthalmologists (LT and RDA).

It was indeed possible that, if some artifacts had similar signal and spatial feature of nonperfused regions, to falsely classify an area as ischemic.

### Statistical Analysis

A Shapiro-Wilks test was performed to evaluate the departure from normal distribution. Quantitative variables were summarized as mean and standard deviation. Categorical variables were summarized as frequency and percentage. Two-way analysis of variance for repeated measures was performed to evaluate the effect of time, treatment and their interaction on study variables during follow-up. Pearson coefficient was computed to evaluate changes in time of the parameters as well as correlation among them. For all analyses, a *P* value of less than 0.05 was considered as statistically significant. Statistical analysis was performed using IBM SPSS Statistics v20.0 software (SPSS Inc. Chicago, IL).

The reproducibility of the results, obtained with the separate revisions of the automatic segmentation by the two ophthalmologists, was compared by estimating the intraclass correlation coefficient. The intraclass correlation coefficient was evaluated considering all the computed quantitative metrices after an among patients’ normalization (z-score).

### Main Outcome Measures

The main outcome measures considered in the study were the following:

1) Nonperfusion area changes after IDI at SCP and DCP level from WFOCTA scans; and
2) Retinal vessel density changes after IDI of SCP and DCP from WFOCTA scans.

## Results

**Figure 2.**
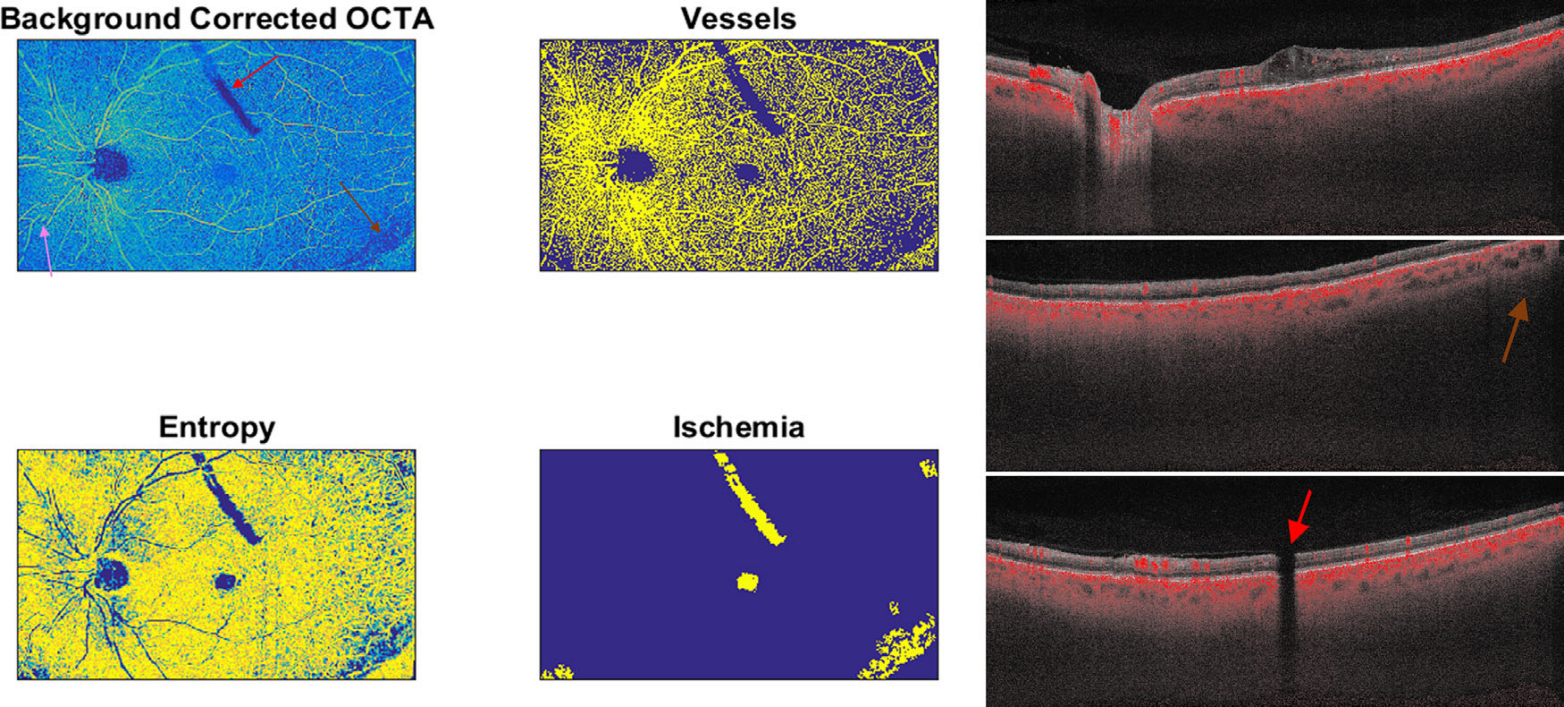
A representative OCTA scan that was excluded owing to many artifacts, such as a shadowing artifact related to dexamethasone implant clearly visible in the superior part of the scan (*red arrow*), motion artifacts with misalignment of retinal vessels (*pink arrow*), and low intensity signal in the peripheral edges of the scan because the retina was not within the focal range on the OCT B scan (*brown arrow*).

Among all scans acquired for whole follow-up (*N* = 224 scans), 15 scans showed segmentation errors. These scans were manually edited by the two retinal specialists as reported in methods section. After segmentation correction they were used in step one. In contrast, two scans could not be used in step one because of the presence of significant artifacts (Fig. 2).

A total of 28 eyes with DME (non-PDR, 23 eyes; PDR, 5 eyes) met the required image quality criteria and were considered in the analysis. Demographic and clinical data are reported in Table 1.

**Table 1.** Patient Demographic and Clinical Data at T0

Variable	Overall Diabetic Group (28 eyes)
Age (years), mean ± SD	54.7 ± 13.8
Sex, *n* (%)	
Male	16 (57.14)
Female	12 (42.86)
Diabetes duration (years), mean ± SD	15.2 ± 8.1
HbA1c (%), mean ± SD	7.2 ± 0.7
Hypertension, *n*	12
Hyperlipidemia, *n*	4
IOP mm Hg, mean ± SD	14.5 ± 2.54
DR stage, *n*	
NPDR	24
PDR	4
Eye treated, *n*	
Right	16
Left	12

HbA1c, hemoglobin A1c; IOP, intraocular pressure; SD, standard deviation.

**Figure 3.**
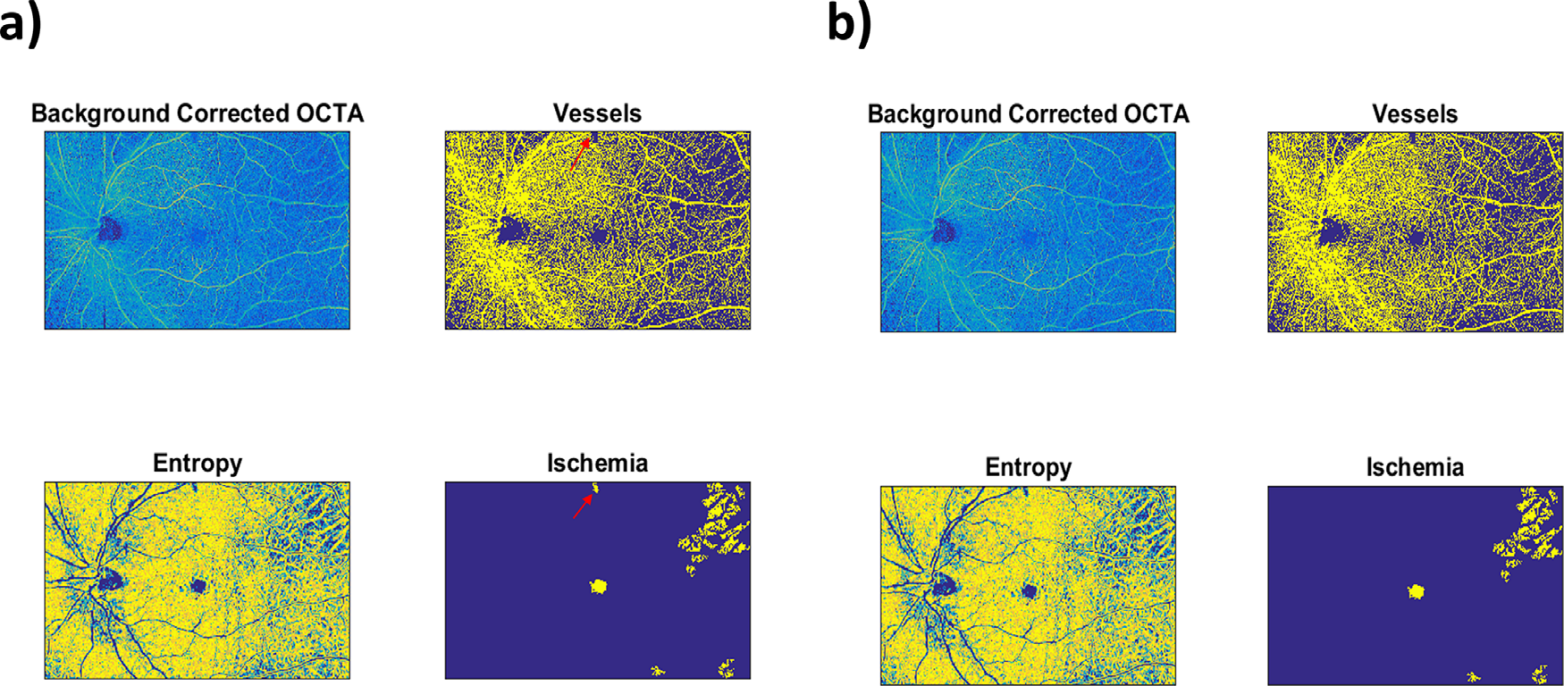
(A) In step two, a little shadowing artifact visible at the superior vascular arcade was considered erroneously as nonperfusion area by the algorithm (*red arrow*). (B) In step three, a manual correction was performed and ischemic areas were recalculated again with the automated algorithm.

In all eyes, retinal nonperfusion areas at SCP and DCP and vessel perfusion density of SCP and DCP were calculated. A high reproducibility of the results when the nonperfusion areas were manually revised by the two independent ophthalmologists was obtained (intraclass correlation coefficient of 0.96). In detail, after the review of nonperfusion areas analysis, only two scans needed a slight manual change by the two graders eliminating little shadowing artifacts that the algorithm considered erroneously as ischemic as represented in the Figure 3.

**Figure 4.**
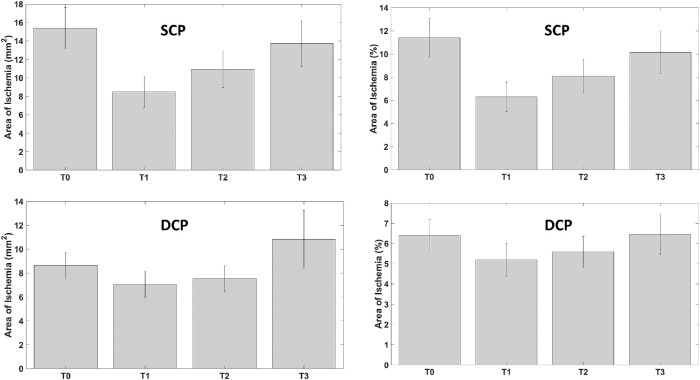
Retinal nonperfusion retinal capillary areas modification during follow-up of SCP (*top*, in square millimeters and in percentage) and of DCP (*bottom*, in square millimeters and in percentage).

**Figure 5.**
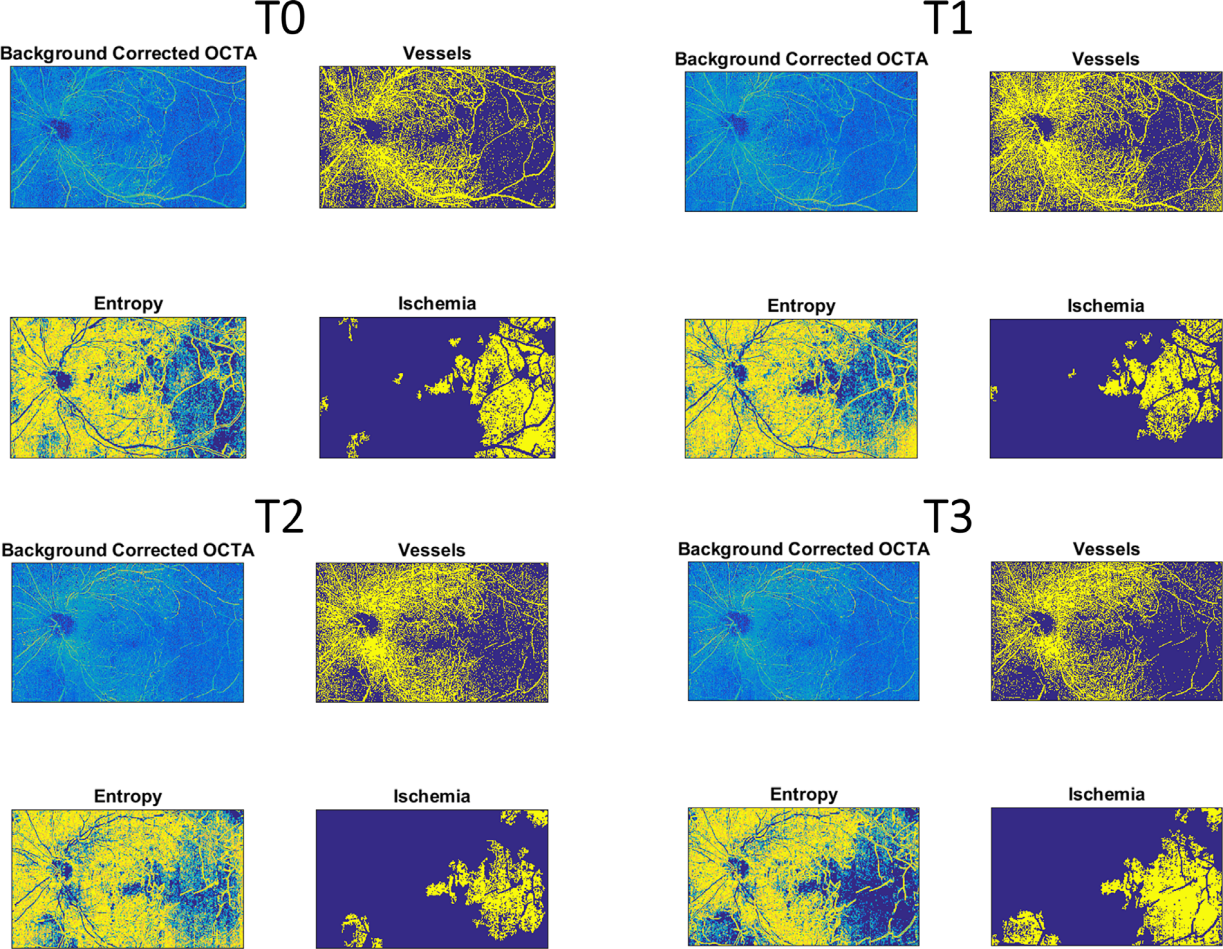
Retinal nonperfusion retinal capillary areas modification of SCP in a single patient after IDI during follow-up.

SCP showed a statistically significant decrease of ischemic areas 1 month after IDI (*P* = 0.04) and slightly increased not significantly thereafter (*P* = 0.15) (Fig. 4; Fig. 5; Table 2).

**Table 2.** Whole Ischemic Areas and Vessel Density Changes at SCP and DCP During Follow-up

	Overall Group						
					*P* Value	*P* Value	*P* Value
Variable	T0	T1	T2	T3	(T1 vs T0)	(T2 vs T1)	(T3 vs T2)
Whole ischemic area at SCP (mm^2^) (%), mean ± SD	15.40 ± 2.23 (11.4%)	8.50 ± 1.71 (6.3%)	10.92 ± 1.97 (8.1%)	13.71 ± 2.46 (13.7%)	0.041	0.460	0.316
Vessel density at SCP (%), mean ± SD	35.30%	38.00%	37.85%	36.04%	0.426	0.860	0.198
Whole ischemic area at DCP (mm^2^) (%), mean ± SD	8.63 ± 1.08 (6.39%)	7.03 ± 1.10 (5.2%)	7.54 ± 1.05 (5.6%)	10.82 ± 2.44 (8.0%)	0.318	0.769	0.1938
Vessel density at DCP (%), mean ± SD	30.87%	29.59%	30.44%	29.25%	0.235	0.422	0.178

SD, standard deviation.

In detail, whole ischemic areas at SCP were 15.40 ± 2.23 mm^2^ (11.4%) at T0; 8.50 ± 1.71 mm^2^ (6.3%) at T1; 10.92 ± 1.97 mm^2^ (8.1%) at T2; and 13.71 ± 2.46 mm^2^ (10.2%) at T3.

DCP did not show a significant reduction of ischemic areas during whole follow-up (*P* = 0.31; Fig. 4, Table 2). In detail, whole ischemic areas at DCP were 8.63 ± 1.08 mm^2^ (6.39%) at T0; 7.03 ± 1.10 mm^2^ (5.2%) at T1; 7.54 ± 1.05 mm^2^ (5.6%) at T2; and 10.82 ± 2.44 (8.0%) mm^2^ at T3.

**Figure 6.**
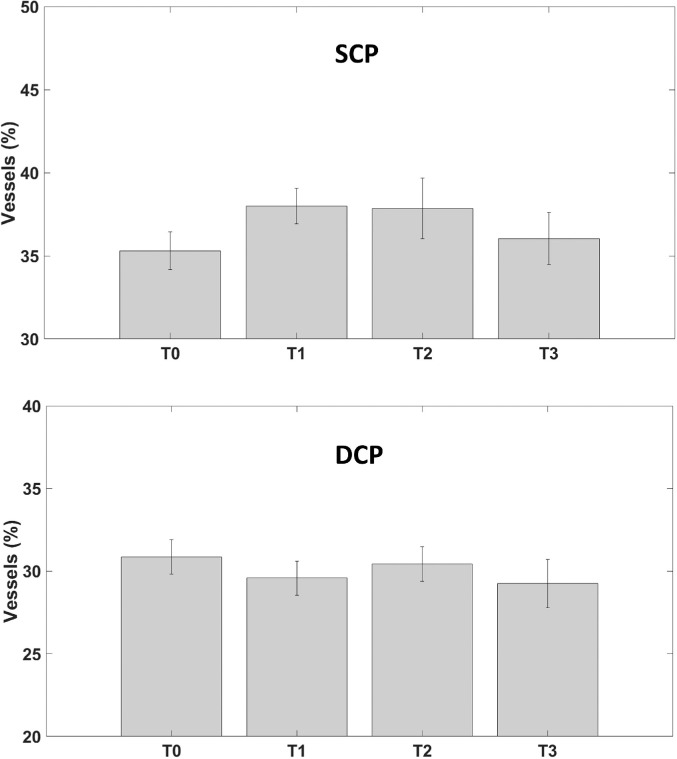
Vessel density modification during follow-up of SCP (*top*, in percentage) and of DCP (*bottom*, in percentage).

SCP vessel density increased not significantly at T1 and then start to decrease, not reaching preoperative values at T3 (*P* = 0.29, Fig. 6, Table 2).

The whole vessel density of SCP was 35.30% at T0, 38.00% at T1, 37.85% at T2, and 36.04% at T3.

DCP vessel density did not change significantly during follow-up (*P* = 0.73; Fig. 6, Table 2). The whole vessel density of DCP was 30.87% at T0, 29.59% at T1, 30.44% at T2, and 29.25% at T3.

## Discussion

Our study evaluated the retinal capillary nonperfusion areas modification at SCP and DCP after IDI in patients with diabetes with DME using a WFOCTA scan of 15 × 9 mm. Also, we measured flow features through quantification of vessel density of both retinal plexuses in the whole retinal images. Overall, we found a reduction of ischemic areas at T1 with a slightly not significantly increase thereafter not reaching preoperative values. Correspondingly, the whole vessel density of SCP slightly increased not significantly from T0 to T1, and then decreased during follow-up. Retinal capillary nonperfusion areas and retinal vessel density at the DCP did not change significantly.

Some studies investigated peripheral ischemic areas in diabetic eyes using wide-field OCTA.^17^^–^^20^

Hirano et al.^19^ investigated superficial and DCPs using small field and WFOCTA and found a reduction in retinal perfusion with worsening DR. Better prediction of DR was evidenced when using small scan sizes compared with widefield scans.^19^

Mastropasqua et al.^16^ evaluated the correlation between central and midperipheral perfusion parameters using WFOCTA. A significant positive correlation was found between macular area and periphery in terms of perfusion density of DCP and choriocapillaris both in diabetics without DR and in diabetic with different stage of DR.^17^

The behavior of retinal ischemia in DR after intravitreal treatment has been evaluated either with FA or OCTA giving new insight to DR pathogenesis.

The BOLT study was a prospective, randomized trial that provided a quantitative analysis of macular perfusion status before and after bevacizumab using FA, showing not statistically significant modification after treatment.^7^

Campochiaro et al.^8^ showed that monthly injections of ranibizumab can slow, but not completely prevent, retinal capillary closure in patients with DME. The data from that study were obtained using FA. 

Bonnin et al.,^9^ in a retrospective interventional study found an improvement of the DR severity score in patients with DR treated with anti-VEGF without retinal reperfusion on ultra-widefield FA.

Instead, a pilot study, conducted by Querques et al.^10^ investigated the effect of dexamethasone intravitreal implant on peripheral ischemia in patients affected by DME using ultra-widefield FA. Dexamethasone implant was effective not only in decreasing breakdown of the blood–retinal barrier, but also in improving ischemic index in all patients.

A post hoc analysis of the phase III VISTA study in patients with DME showed that intravitreal aflibercept with regular dosing could delay worsening of retinal perfusion associated with DR and also may be improve retinal perfusion in some cases by decreasing zones of retinal nonperfusion.^11^

Recently, Couturier et al.^21^ found no reperfusion of vessels or capillary network in nonperfusion areas in patients with DR after 3 anti-VEGF injections using both swept source WFOCTA and ultra-widefield FA. 

In our study, we found a significant decrease in retinal nonperfusion capillary areas at 1 month after IDI in the SCP, although no significant modification was found in the DCP. In addition, the perfusion density of SCP and DCP increased not significantly at T1 compared with T0 values in the whole WFOCTA scan.

In accordance with some evidence from the literature our findings suggest that areas of ischemia can possess viable, salvageable tissue with the potential to reperfuse.

In animal studies, it has been demonstrated that, in DR, increased expression of VEGF plays a major role in the progression of nonperfusion. High levels of VEGF in the retina stimulate recruitment and adhesion of leukocytes (leukostasis), resulting in plugging of large and small retinal vessels. Increased leukostasis is related to increased nuclear factor κB transcriptional activity in retinal endothelial cells and in the retina related to high VEGF levels whose product is nuclear factor κB–responsive gene Vcam1. Thus, the proposed hypothesis for retinal reperfusion after turning off VEGF expression or after intraocular injection of a VEGF-neutralizing protein decreases leukostasis, allowing closed vessels to reopen.^27^

An alternative hypothesis after anti-VEGF treatment that there is a decrease in hyperpermeability, pericyte restoration, and normalization of the basement membrane. This normalized basement membrane provides a scaffolding for regrowth of retinal microvasculature.^28^

In our study, we found a lower reperfusion in DCP compared with SCP, both showed by lower reduction of ischemic areas and increase of vessel density during follow-up.

Campochiaro et al.^8^ demonstrated the retinal nonperfusion in patients with DR is not always reversible after anti VEGF treatment. Perhaps, in the process of vascular damage there is an early phase in which vascular occlusion would be reversible.

Several previous studies showed that DCP is the early target of vascular changes in DR and is more compromised compared with SCP.^14^^–^^16^^,^^29^

We can argue that the more prolonged damage of DCP compared with SCP made some of the nonperfusion areas of this plexus not reversible.

Dexamethasone implant showed a strong effect 1 month after treatment and subsequent gradual loss of effectiveness. It is known that IDI releases a decreasing quantity of corticosteroids in time-dependent fashion. In fact, pharmacokinetic data from a 9-month study of the dexamethasone implant in primate eyes showed that dexamethasone implant is present at high concentrations in the vitreous humor and retina for up to 60 days and can be maintained for 6 months after administration.^30^^–^^32^

The drug's pharmacokinetic could explain the greater effect immediately after implant and the gradual loss of effectiveness in the following months.

The molecular mechanism by which currently available therapies normalize retinal blood vessels and reduce the ischemic areas in DR should be better explored.

The strength of this study is the semiautomated calculation of ischemic areas. A limitation of this approach is that large vessels (with a section >5 pixels) tend to provide regions of low entropy; however, the regions of large vessels can be easily masked based on the thresholded vessel image.

Presently, OCT angiography allows the study of the retinal vascular bed and discriminates between superficial and deep retinal vascular plexa. Furthermore, the development of automated algorithms provides quantitative measures of retinal flow parameters.

The rationale behind the implementation of a semiautomated approach to perform OCTA segmentation of nonperfusion areas was driven by two main reasons.

The first, more practical reason, is indeed the time-saving aspect. In fact, similarly to our work, Querques et al.^10^ analyzed peripheral ischemic areas that were manually delineated and then nonperfusion areas were manually calculated. This is very time consuming. Conversely, our study aimed at implementing a semiautomated algorithm to automatically quantify areas of nonperfusion and to easily adjust the results through a quick expert analysis of the images.

The second reason is moving toward a more objective way of analyzing OCTA images. Indeed, a complete objectivation of the approach is only reached when a fully automatic procedure is implemented; however, the presented algorithm is a work in progress toward that goal. Automation and subsequent objectivation of the results would allow for an easier comparison among results obtained across different clinical studies.

All these technologies allow a great step forward for the study of DR. These findings should be considered as a starting point to promote prospective clinical trials to better analyze the effects intravitreal treatment on recovering retinal nonperfusion and avoiding worsening of vascular damage in patients with DR.
